# Enzymatic Hydrolysis of Sugarcane Bagasse in Aqueous Two-Phase Systems (ATPS): Exploration and Conceptual Process Design

**DOI:** 10.3389/fchem.2020.00587

**Published:** 2020-07-31

**Authors:** Bianca Consorti Bussamra, Paulus Meerman, Vidhvath Viswanathan, Solange I. Mussatto, Aline Carvalho da Costa, Luuk van der Wielen, Marcel Ottens

**Affiliations:** ^1^Department of Biotechnology, Delft University of Technology, Delft, Netherlands; ^2^Development of Processes and Products (DDPP), University of Campinas, Campinas, Brazil; ^3^Novo Nordisk Foundation Center for Biosustainability, Technical University of Denmark, Kongens Lyngby, Denmark; ^4^Bernal Institute, University of Limerick, Limerick, Ireland

**Keywords:** sugarcane bagasse, product inhibition, enzymatic hydrolysis, extractive process, aqueous two-phase systems (ATPS)

## Abstract

The enzymatic conversion of lignocellulosic material to sugars can provide a carbon source for the production of energy (fuels) and a wide range of renewable products. However, the efficiency of this conversion is impaired due to product (sugar) inhibition. Even though several studies investigate how to overcome this challenge, concepts on the process to conduct the hydrolysis are still scarce in literature. Aqueous two-phase systems (ATPS) can be applied to design an extractive reaction due to their capacity to partition solutes to different phases in such a system. This work presents strategies on how to conduct extractive enzymatic hydrolysis in ATPS and how to explore the experimental results in order to design a feasible process. While only a limited number of ATPS was explored, the methods and strategies described could easily be applied to any further ATPS to be explored. We studied two promising ATPS as a subset of a previously high throughput screened large set of ATPS, providing two configurations of processes having the reaction in either the top phase or in the bottom phase. Enzymatic hydrolysis in these ATPS was performed to evaluate the partitioning of the substrate and the influence of solute partitioning on conversion. Because ATPS are able to partition inhibitors (sugar) between the phases, the conversion rate can be maintained. However, phase forming components should be selected to preserve the enzymatic activity. The experimental results presented here contribute to a feasible ATPS-based conceptual process design for the enzymatic conversion of lignocellulosic material.

## Introduction

The search for alternatives to replace fossil fuels by renewable energy has been seen as a major necessity in modern times (Passoth and Sandgren, 2019). In order to reach the requirements established by the Paris Agreement in 2017, net zero greenhouse gases emission must be achieved by 2050 (Pye et al., 2017). Sun, wind, and lignocellulosic residues can be sources of renewable energy (Goldemberg and Teixeira Coelho, 2004). However, unlike wind and solar energy, biomass (lignocellulosic residues) can provide a carbon source not only for energy (fuel) production, but also for the production of a wide range of renewable products (Straathof, 2014). Sugarcane bagasse is the most abundant lignocellulosic residue in Brazilian agriculture. Among the 1.8 billion tons of sugarcane processed annually in the world, Brazil is the first producer, holding 41% of the world production. This amount yields ~105 million tons of the residue (sugarcane bagasse) per year (UN Food and Agriculture Organization, 2017).

The enzymatic conversion of sugarcane bagasse to obtain monomers of glucose usually presents a limited efficiency as a consequence of several factors such as the cellulose crystallinity, product inhibition, and enzyme degradation (Gupta et al., 2016). There are several research lines cooperating to improve the utilization of lignocellulose as a raw material to the production of biofuel and chemicals. Pre-treatment of the biomass is necessary to expose the cellulose component to the cellulases. In order to establish a good balance between the decrease of the biomass recalcitrance to enzymatic hydrolysis and disadvantages of each pre-treatment, several types of processes have been investigated: physicochemical, chemical using acids and alkalis, and solvent extraction (Liu et al., 2019). Biological pre-treatments have been proposed as an alternative to chemical ones, mainly because of the reduction of toxic compounds and fermentation inhibitors generation (Sindhu et al., 2016), and improvement in the subsequent enzymatic hydrolysis (Vaidya and Singh, 2012; Singh et al., 2016). The inhibition on cell growth and metabolism, which leads to reduction of product of interest formation in fermentation, can be caused by substances present in the plant composition or released during pre-treatment and/or hydrolysis processes. Among many strategies to mitigate this product-induced inhibition of microbes, studies highlight the use of stabilizing substances and conditions, stream selection, and product removal (Cray et al., 2015). In the spectrum of the biocatalysis, the enzyme degradation can be related, for instance, to chemical reaction with lignin (Newman et al., 2013). The development of new biocatalysts, such as accessory enzymes, and use of additives (Donaldson et al., 2014; Vaidya et al., 2014; Fahmy et al., 2019) have been identified as potential fields to promote more efficient and tolerant enzymatic reactions. Moreover, the synergy of fungal enzymes and the design of rational cocktails have provided improved hydrolysis performance (Bussamra et al., 2015; Cameron et al., 2015; Gupta et al., 2016). However, few studies question the conventional process to conduct the enzymatic hydrolysis of cellulose to monomers, e.g., the optimization of reaction conditions, reactor design, enzyme recycling, and recovery strategies. The main drawback regarding the enzymatic hydrolysis is the product inhibition of the enzymes (Bezerra and Dias, 2005; Gupta et al., 2016). In other words, the higher the glucose concentration, the less efficient the process is. By addressing this need, a more efficient enzymatic hydrolysis could be performed, reducing the costs of the process, enhancing the yield, and contributing to establish biomass as feedstock for the production of renewables.

In order to overcome the challenge of product inhibition during enzymatic hydrolysis, some solutions have been suggested in literature, including: simultaneous saccharification and fermentation (SSF) (Mohagheghi et al., 1992), development of glucose tolerant enzymes (Cao et al., 2015), partial cellulose hydrolysis (direct use of lignocellulose-derived sugars by the microorganisms) (Chen, 2015), and *in situ* product removal (Hahn-Hägerdal et al., 1981; Yang et al., 2011). Extractive processes such as SSF indicates a positive effect on cellulose hydrolysis because the inhibition by ethanol is less harmful to enzymes than the one caused by cellobiose (Bezerra and Dias, 2005). However, ethanol inhibition still exposes a potential problem in the SSF (Wu and Lee, 1997). In order to remove the inhibitor, ATPS can be applied as a strategy to separate the product and the enzymes. ATPS are formed by two immiscible components dissolved in water (Benavides et al., 2011). These components, named as phase forming components (PFC), can be polymers, salts (Van Sonsbeek et al., 1993), or ionic liquids (Freire et al., 2012). Above a critical concentration of the PFC, the system presents two phases in which molecules (solutes) can be unevenly partitioned in accordance to the system composition (Baskir et al., 1989).

Even though extractive processes based on *in situ* product removal have been extensively applied to enzymatic reactions (Ferreira et al., 2018) and/or fermentation (Hahn-Hägerdal et al., 1981; Kulkarni et al., 1999), to the extent of our knowledge, enzymatic hydrolysis in biphasic systems composed by salt-polymer is still rarely reported. This work aims to unlock the potential of a novel and extractive process to conduct the enzymatic hydrolysis of lignocellulosic materials. In this study, we evaluated the efficiency of performing the extractive reaction by ATPS composed by polyethylene glycol and the salts potassium citrate and magnesium sulfate. In the referred systems, glucose and the reactive phase (bagasse and enzymes) can be separated to prevent product (glucose) inhibition. Moreover, we showed how experimental results at lab scale and parameters of the process are interconnected, providing information on technical conditions to further design a new industrial process for lignocellulosic conversion.

## Methods

### Research Design

**Figure 1:**
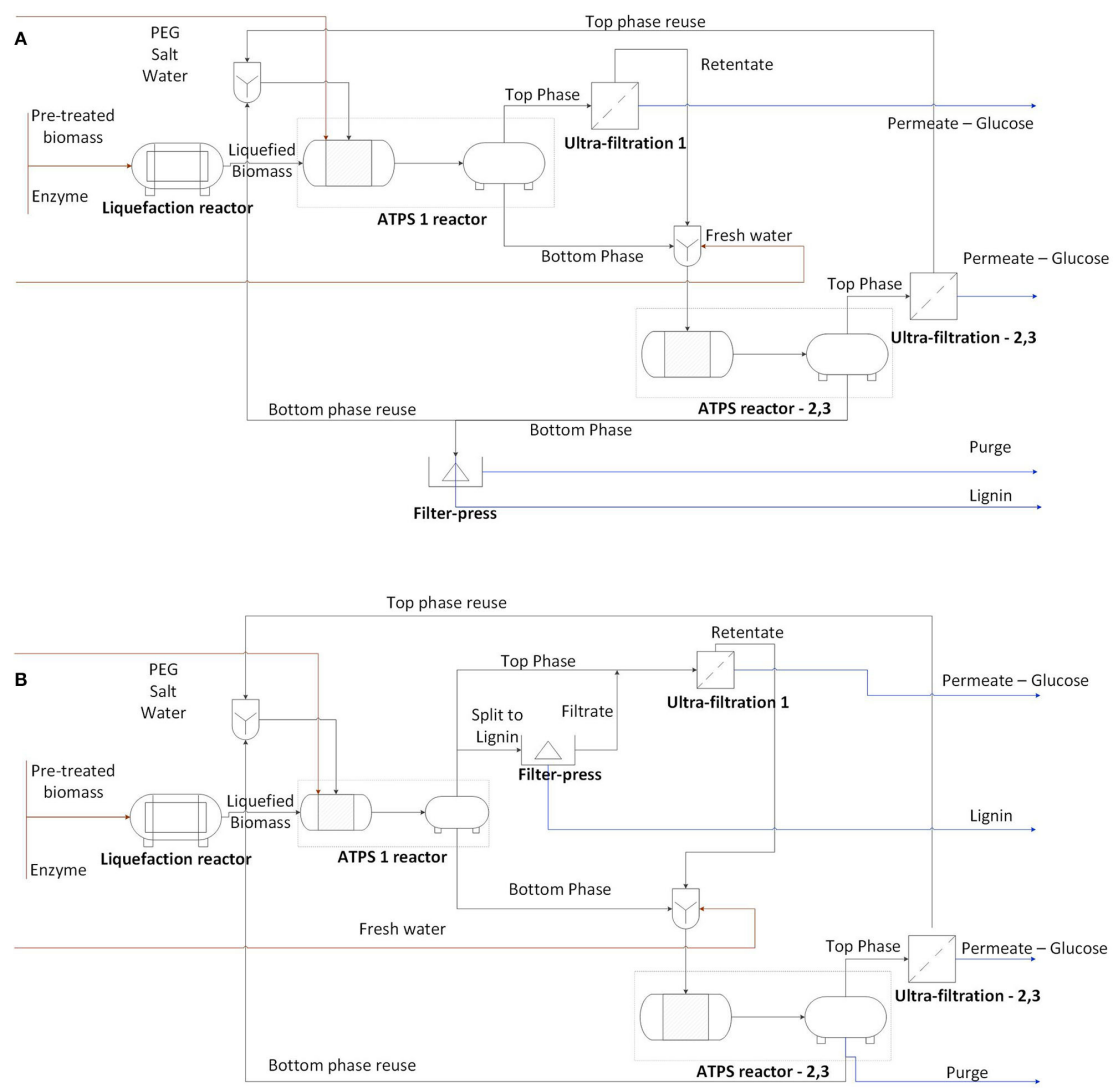
Conceptual process design for **(A)** hydrolysis in bottom phase (schematic representation of an ATPS process for magnesium sulfate and PEG 6000) or **(B)** hydrolysis in top phase (schematic representation of an ATPS process for potassium citrate and PEG 6000). The numbers represent the how many cycles each unit of operation operates, which can vary for each ATPS and desired efficiency and recovery of the process. Considering an ATPS step with both mixing and separation compartments, two vessels are presented per ATPS unit operation.

In this work, the enzymatic hydrolysis of sugarcane bagasse in ATPS was explored and two processes were designed. According to the partitioning of bagasse and solutes in the phases, the reaction and extractive phases can alter. Based on that, two approaches were discussed: hydrolysis occurring in the bottom phase (Figure 1A) and in the top phase (Figure 1B). The process design for each approach was developed according to experimental data and literature evidences. However, the quantitative evaluation of each unit of operation is out of the scope of this work.

Experimental data provided information for the design of ATPS reactor and ultrafiltration unit operations. The linkage between the experimental results and their usage into the process design is presented in Table 1. The strategy suggested for sugar recovery was based on literature evidence. The findings regarding the behavior of sugarcane bagasse hydrolysis in ATPS, connected to the potential of system components recycle, yielded insights on how feasible this extractive conversion could be.

**Table 1:** Experiments performed to enable a reasonable process design parameters definition.

**Experiment**	**Type of parameter to be defined and the respective unit operation in the process design**
Salt type and polymer molecular weight influence on bagasse partition	ATPS reactor: reaction phase (top or bottom)
Density of PFC before and after their adsorption to bagasse	ATPS reactor: addition mode of PFC to the bagasse
Enzymatic load influence on the concentration of free enzymes (not adsorbed) on conventional hydrolysis (kinetics) and specific enzymatic activities in the supernatant	Ultrafiltration: feasibility to recycle enzymes in terms of available amount of free proteins
TLL (tie line length), salt type and polymer molecular weight influence on the partitioning of proteins and sugars (K_s_/K_p_)	ATPS reactor: concentration of PFC in reaction
TLL and time of ATPS hydrolysis (kinetics) influence on sugar release	ATPS reactor: concentration of PFC and residence time of reaction
Rate of enzymatic hydrolysis (conventional and ATPS)	ATPS reactor: feed (flow) rate of substrate in order to maintain constant water-insoluble substrate (WIS) in a continuous operation mode and to achieve the desired yield
Binodal curves and tie lines data	ATPS reactor: estimation of outlet concentrations of top and bottom phases given an inlet composition of ATPS
Partitioning of specific enzymatic classes in ATPS (top and bottom)	Ultrafiltration: feasibility to recycle enzymes in terms of enrichment of one specific enzymatic class in continuous operation

The conversion of lignocellulosic biomass into sugars was catalyzed by the enzymes presented in the commercial cocktail Cellic CTec 2 (Novozymes, Bagsværd, Denmark). In order to create the extractive environment, ATPS formed by polymer and salts were applied to conduct the reaction. In the explorative investigation of ATPS applied to lignocellulosic conversion, topics such as the adsorption of phase forming components to the bagasse fibers and the influence of enzyme load on the hydrolysis were approached. Sequentially, the potential of recycling the enzymes in a continuous process was evaluated according to the distribution of specific enzymes among the phases and the adsorption of specific enzymatic activities to the bagasse. To complete the definition of parameters to further design the extractive process, the continuous recovery of sugar (glucose) from the system was theoretically analyzed.

### Materials

The Brazilian Biorenewables National Laboratory (LNBR) provided the sugarcane bagasse: hydrothermally pre-treated (at 190°C, 10 min) followed by delignification (at 100°C, 1 h, and 1% NaOH). Although the presence of lignin does not influence the decrease in conversion with increasing solid loads (Modenbach and Nokes, 2013), this bagasse treatment was selected due to the fact that lignin could interfere in the enzyme performance and, consequently, in the product inhibition study. The bagasse, composed of 79.2% ± 0.9 cellulose, 2.6% ± 0.1 hemicellulose, 12.6% ± 0.3 lignin, and 6.2% ± 0.2 ash [composition determination as defined by Sluiter et al. (2016)], presented a wet basis humidity of 7.6% and was milled at 0.08 mm mesh. The milling process occurred at the speed of 104 rotations per minute (RPM) (Fritsch, Pulverisette 14).

Polyethylene glycol (PEG) with molar mass 2000 g/mol (PEG 2000) and 4000 g/mol (PEG 4000) were purchased from Merck (Darmstad, Germany). PEG 6000 (6000 g/mol) and magnesium sulfate heptahydrate (MgSO_4_·7H_2_O) were acquired from J.T. Baker (Fisher, New Jersey, USA). Sodium carbonate (Na_2_CO_3_) and 3,5-Dinitrosalicylic acid (DNS), both used for enzymatic activities assays, anhydrous glucose, and potassium citrate tribasic monohydrate (K_3_C_6_H_5_O_7_·H_2_O) were supplied by Sigma Aldrich (Taufkirchen, Germany). The pH was adjusted by adding sodium hydroxide (4 M) or hydrogen chloride, both purchased from Merck. Citrate buffer 50 mM was prepared according to Adney and Baker (2008).

Stock solutions were prepared by dissolving the respective PEG molecular weight or salt in double distilled deionized water (Milli-Q water), in order to obtain the following stock solutions: 38% w/w PEG 2000, 40% w/w PEG 4000, 39% w/w PEG 6000, and 40% w/w magnesium sulfate or potassium citrate solutions.

### ATPS Formation (Equilibrium and Separation)

The selection of ATPS composition (salt type and polymer molecular weight) and concentration (tie line length–TLL) were based on data previously published by Bussamra et al. (2019). The ATPS, respective TLL, and experiment to which they were applied and motivation are presented in Table 2.

**Table 2:** Connection of the selected ATPS to respective application in experiments.

**System**	**Phase forming components**	**TLL (%)**	**Experiment**	**Motivation**
ATPS 1	Potassium citrate and PEG 2000	12.8	Glucose and protein partition	Low TLL, less inhibition of enzymes by PFC. Less viscous system compared to PEG 6000.
ATPS 2	Potassium citrate and PEG 6000	15.3	Glucose and protein partition; Hydrolysis kinetics	Lowest TLL reported by Bussamra et al. (2019) for this system composition. Enzymes could be more active compared to ATPS 3.
ATPS 3	Potassium citrate and PEG 6000	23.7	Glucose and protein partition	High precipitation of proteins.
ATPS 4	Magnesium sulfate and PEG 2000	20.5	Glucose and protein partition	High *K*_*sugar*_. Enzymes could be more active compared to ATPS 5 (ATPS 4 is less concentrated and less viscous).
ATPS 5	Magnesium sulfate and PEG 6000	30.5	Glucose and protein partition; Hydrolysis kinetics	High sugar selectivity (*K*_*sugar*_/*K*_*protein*_).
ATPS 6	Potassium citrate and PEG 6000	34.2	Liquefaction following hydrolysis	Lowest TLL to provide high *K*_*protein*_ (Bussamra et al., 2019).

The ATPS 1–5 were evaluated for the partitioning of glucose and proteins at a fixed concentration of each solute and without the presence of substrate. Because ATPS 6 was not favorable to conduct hydrolysis, the partitioning of solutes was not evaluated at this composition. Systems were formed by addition of the required amount of PEG and salt stock solutions to an aqueous solution of 5 mL total volume. Sugar stock solution (800 g/L, prepared in Milli-Q water) was added to a final concentration of 90 g/L in the system. The volume of enzymes (29.5 μL) was calculated considering a hypothetical solid load of 10% WIS and an enzyme load of 10 FPU/g bagasse. This amount of enzymatic cocktail corresponded to a protein concentration of ~0.92 mg/mL. The systems were incubated for 1 h at 50°C and 250 rpm in orbital shaker. Phase separation was promoted by centrifugation at 4,000 rpm, 40°C for 30 min (Eppendorf 5810R Multipurpose Centrifuge®). Top phase was withdrawn using an automatic pipet and bottom phase via 2 mL syringe coupled with appropriated needle. All systems were prepared in duplicates. The partition coefficient in a two-phase system was determined as the ratio of solute concentration in the top phase to that in the bottom phase (Li et al., 2002).

### Adsorption of Phase-Forming Components to Sugarcane Bagasse

Predetermined amounts of bagasse (5% WIS) were emerged for 3 h at 250 rpm and 50°C in four different solutions of phase-forming components (final volume 15 mL): magnesium sulfate (40% w/w), potassium citrate (40% w/w), PEG4000 (40% w/w), and Milli-Q water. Subsequently, the samples were centrifuged at 1300 rpm, 40°C for 30 min (Eppendorf 5810R Multipurpose Centrifuge®). The bagasse was separated from the supernatant and the wet basis humidity was determined using a moisture balance (Sartorius Ma35). The density of both the stock solutions of phase forming components and the supernatant liquids, measured using a pycnometer, was 5.113 cm^3^ (Blaubrand, Germany). The comparison in density of both solutions (stock and supernatant) indicates whether there is a preferential adsorption of phase forming components to sugarcane bagasse in relation to water (for instance, a less concentrated supernatant illustrates a preferential adsorption of the phase-forming component to the substrate).

### Conventional and ATPS Enzymatic Hydrolysis

Conventional hydrolysis occurs in the presence of citrate buffer 50 mM, pH 4.8, with 0.02% sodium azide. The conventional hydrolysis kinetics at solid load 10% WIS were performed at enzyme loads 10, 20, and 40 FPU/g bagasse at a reaction volume of 30 mL. Samples from conventional hydrolysis were centrifuged at 4,000 rpm, 4°C, for 10 min (Eppendorf 5810R Multipurpose Centrifuge®). Supernatants were collected for both protein and glucose quantifications. Conversion of biomass (*x*) was calculated according to the following formula, taking into account the amount of soluble sugars in the liquid phase after hydrolysis (*S*_*g*_, in glucose equivalent concentration; *V*_*H*_, the volume of the solution where the hydrolysis was performed; and the correction factor for hydration 0.9, in order to correct for the water molecule added upon hydrolysis [Selig et al., 2008]), and the initial amount of glucose equivalent in the solid cellulose sample before hydrolysis (*W*, the weight of dry lignocellulosic sample, and *F*_*g*_, the fraction of cellulose in the lignocellulosic sample):

(1) $$\begin{array}{l} \text{x }=\;\left(\frac{{\text{S}}_{\text{g}}\cdot {\text{V}}_{\text{H}}}{{\text{W}}_{dry\;bagasse}\cdot {\text{F}}_{\text{g}}}\right)\cdot 0.9\cdot 100\% \end{array}$$

ATPS hydrolysis was conducted at ATPS 2 and 5, as presented in Table 2. The phase-forming components (Milli-Q water and salt and PEG stock solutions) were mixed to achieve a volume ratio of 1:1, in a reaction volume of 15 mL according to the respective TLL. The bagasse was added to the system under agitation at a solid load of 10% WIS. Lastly, sodium azide and enzyme were added to a load 0.02% and 20 FPU/g bagasse, respectively. For each time evaluated, a unique system in duplicate was prepared once the withdrawal of top and bottom phases requires the discontinuation of the reaction and centrifugation of the system. The centrifugation occurred for 30 min, at 4,000 rpm and 40°C (Eppendorf 5810R Multipurpose Centrifuge®). All hydrolysis reactions were performed at 50°C and 250 rpm in orbital shaker.

### Liquefaction Followed by Hydrolysis

For the hydrolysis following the liquefaction, the ATPS were formed by the addition of 9.9 mL of salt (potassium citrate 40% w/w) and 10.1 mL of polymer (PEG 6000 39% w/w) to 6 mL of liquefied bagasse (20% WIS and 10 FPU/g bagasse, for solid load and enzyme load, respectively, in citrate buffer 50 mM pH 4.8, hydrolyzed for 24 h). Considering the liquefied bagasse volume being the solvent of the ATPS, the hydrolysis was performed at the TLL 34.2% and volume ratio of 1. However, the ATPS was more concentrated because part of the liquefied volume was occupied by cellulose, lignin, ash, and already released sugars. For comparison with a conventional system, 20 mL of buffer (citrate 50 mM pH 4.8) was added to 6 mL of liquefied bagasse.

### Enzymatic Activities

An international unit of enzymatic activity is defined as the amount of enzyme required to produce 1 μmol of product per minute. 1 mM p-nitrophenyl-β-D-glucopyranoside (p-NPG), 1 mM p-nitrophenyl-β-D-xylopyranoside, and 4 mM p-nitrophenyl-β-D-cellobioside (p-NPC) (Sigma–Aldrich, St. Louis, EUA) were the substrates for the activity determining reaction for β-glucosidase, β-xylosidase and cellobiohydrolase, respectively. After 10 min for β-glucosidase and β-xylosidase and 30 min for cellobiohydrolase activity measurement at 50°C, the reactions (comprised of 80 μL of the substrate and 20 μL of the enzyme) were stopped by addition of 1 mol/L sodium carbonate. Absorbance at 400 nm was used to estimate p-NP concentration release (Bussamra et al., 2015).

The total cellulose activity measured according to the filter paper units (FPU) was performed by reducing the NREL methods (Adney and Baker, 2008) 10 times. The sugar release was quantified following the methods suggested by Miller (1959).

### Sugar and Protein Quantification

Sugar and protein present in top and bottom phases were measured according to methodology described by Bussamra et al. (2019). Sugar quantification followed the Megazyme Glucose Reagent assay (Megazyme, Wicklow, Ireland), and no adaptation was needed to ATPS samples. Hydrolysis samples for glucose measurement were boiled for 5 min and 99°C after collected (Eppendorf Thermomixer® C.), in order to deactivate the enzymes. The protocol used for protein quantification, based on Bradford (1976) method and performed using the Coomassie Protein Assay Reagent (Thermo Scientific, USA), required adaptation for ATPS samples, explained in detail in the cited reference (Bussamra et al., 2019). The solute concentrations in g/L were defined per volume of the respective phase being measured.

The uncertainties of the all measurements calculated by calibration curves were estimated according to Barwick (2003), and the errors were propagated accordingly.

### SDS-PAGE Electrophoresis

For the analysis of specific enzymes partition in ATPS, the bottom phase of ATPS 2 (Table 2) was evaluated in SDS-PAGE electrophoresis. To prevent interference of salt and PEG in the method, the bottom phase had the phase formation exchanged by Milli-Q water using a 10.000 MWCO Amicon® Ultra Centrifugal Filters as described by the supplier. Retentate of this operation (protein enriched) was dissolved in Milli-Q water. Top phase could not be evaluated through this method because the 10.000 MWCO membrane was clotted with the high concentration of PEG 6000 in that phase. The samples were diluted $\frac{3}{4}$ times in NuPAGE™ LDS Sample Buffer (4X) (ThermoFisher Scientific, USA), and this mixture was heated at 70°C for 10 min. 12% (w/v) Bis-Tris polyacrylamide gel (NuPAGE™, 1.0 mm, 12-well) (ThermoFisher Scientific, USA) was loaded with 10 μL prepared samples and stained by GelCode™ Blue Safe Protein Stain (ThermoFisher Scientific, USA). Mark12™ Unstained Standard (ThermoFisher Scientific, USA) was used as molecular weight ladder consisting on the following sizes: 200, 116.3, 97.4, 66.3, 55.4, 36.5, 31, 21.5, 14.4, 6, 3.5, and 2.5 kDa. The protein concentration of the bottom phase sample preparation was 0.39 ± 0.02 mg/mL. The maximum protein load in the band was 0.5 μg.

### Theoretical Sequential Partitioning of Sugar and Recovery

The sequential partitioning of sugar in different stages (continuous process) was modeled based on the mass balance of the components in each stage and according to the equilibrium dictated by the partition coefficients for sugar and enzyme in the ATPS and the concentration factor for the ultra-filtration unit. An initial volume ratio of 1 was assumed for equal volume between the top and bottom phases. According to the partition coefficient and volume ratio, the top and bottom phase concentrations and volume ratio were estimated for each stage (cycle).

The concentration factor determined the permeate and retentate concentrations and flow rates. The permeate was assumed to leave the system as glucose, while the retentate was diluted to the original (top) phase volume and recycled back to the ATPS. Thus, in each stage, the concentration of sugar in the retentate was diluted. The calculation was repeated for each stage under the same assumptions. At the simulation presented here, the two different partition coefficients of sugar were assessed (1.5 and 0.71), considering the same concentration factors of 1.5. The result was assessed in terms of sugar recoveries after the ultrafiltration unit operation.

## Results

The evidence from experiments can guide the definition of parameters to design an extractive process to relieve product inhibition. Table 1 contains the defined process design parameters and the correspondent type of experimental data to substantiate the decision-making. The partitioning of components in ATPS depends on the system composition (Benavides et al., 2011), and determines the strategy to recycle system components and recover products. Here, we presented pre-selected ATPS capable to provide different partition coefficients of sugar and enzymes, and how the phase forming components can influence the partitioning of lignocellulosic biomass. The influence of protein load, enzyme adsorption on bagasse, and partition of specific enzymatic activities in conventional hydrolysis brought insight on the design of the ultrafiltration unit operation regarding the recycle of enzymes.

### Selection of ATPS Based on Partition Coefficients of Solutes

The pre-selected systems were previously indicated by Bussamra et al. (2019) as potential ATPS to separate sugar and enzymes. At this work, five systems, described in Table 2, were reproduced to have their partition coefficient measured at higher scale (5 mL). In the design of a process, more important than the partition coefficient itself is the relation between the partition coefficient of solutes aimed to be separated. The relative partitioning of these solutes represents how efficient their separation is among the phases. In all the systems, the partition coefficients of sugar were higher than for proteins.

The strategy designed to recover the product (sugar) in this process, regardless of which phase the bagasse partitions to, suggests the sequential partitioning of sugar to the top phase and the recovery occurring in that phase. Thus, ATPS 2 and ATPS 5 seemed to be the most efficient for that purpose due to the high selectivity for sugar *K*_*sugar*_/*K*_*protein*_ (Table 3).

**Table 3:** Relative partitioning of sugar and protein in five different ATPS (systems defined in Table 2).

**System**	**K_sugar_**	**K_sugar_/K_protein_**
ATPS 1	0.6	1.6
ATPS 2	0.6	2.4
ATPS 3	0.5	2.2
ATPS 4	0.7	1.7
ATPS 5	0.5	5.4

### Exploratory Investigation of ATPS Applied to Lignocellulosic Hydrolysis

Preliminary investigation on influence of parameters inherent to ATPS (e.g., phase forming components adsorption on the bagasse fibers) is of paramount importance to set up coherent hydrolysis experiments.

The phase-forming components influence the bagasse partition in ATPS. After hydrolysis, the systems composed by potassium citrate favored the lignocellulose partition to the top phase, while systems containing magnesium sulfate triggered the partition to the bottom phase (visual inspection of the systems). Figure 1 presents scenarios where the choice of phase forming components determines whether the reactive phase (phase containing the bagasse) is the top or the bottom phase. The PEG molecular weight was closely related to the partition of the bagasse, being a contributor to increasing the partition of bagasse to the top phase. Moreover, bagasse presented a non-selective adsorption of phase forming components. Consequently, the bagasse adsorption of phase forming components does not influence the system composition.

**Figure 2:**
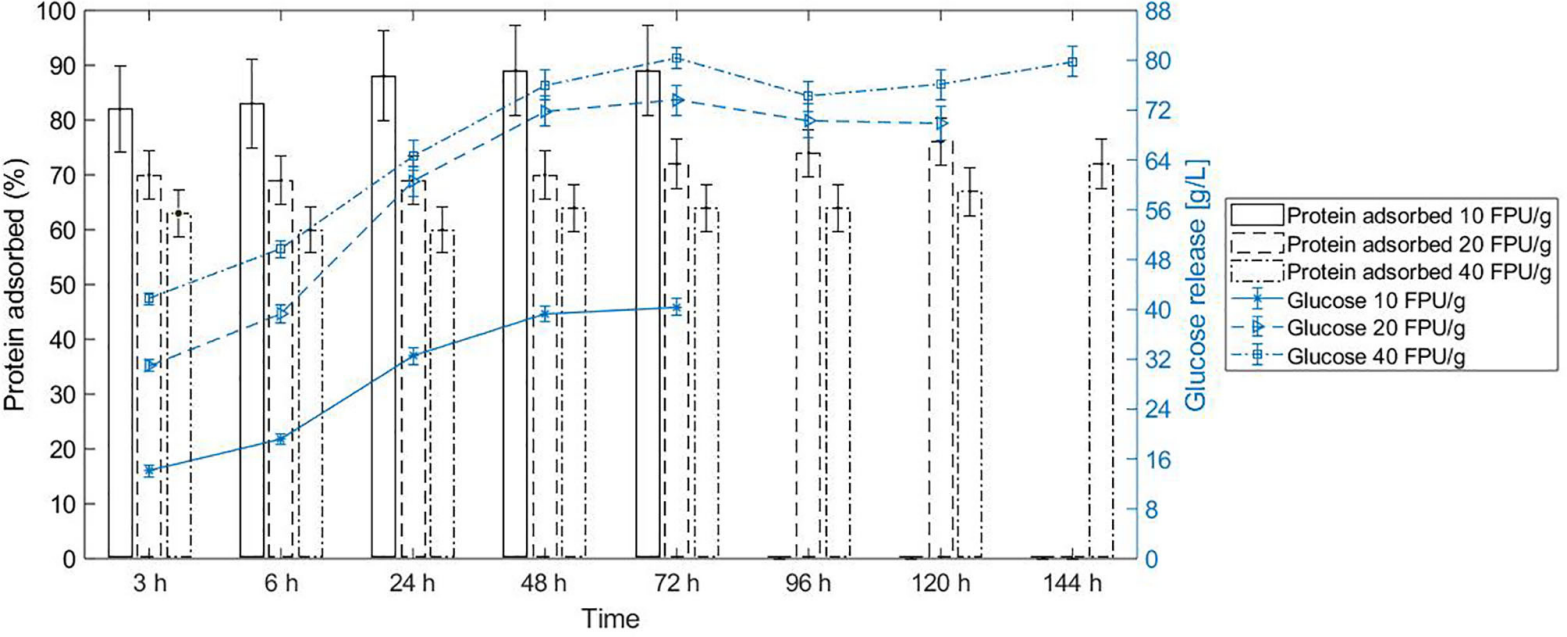
Protein adsorption/desorption does not follow the glucose release in conventional hydrolysis at 10% WIS (solid load), at different enzyme loads of 10 FPU/g bagasse, 20 FPU/g bagasse, and 40 FPU/g bagasse. The bars represent the protein adsorbed (left y axis), and the curve shows the glucose release (right y axis). The conversion of reaction at each enzyme load achieved 47% ± 1.5, 79.4% ± 3.2, and 88.7% ± 2.7, respectively.

The cellulolytic enzymes adsorb to the substrate and consequently partition according to the substrate-enriched phase in the system (Tjerneld et al., 1985). Due to that, the partition of bagasse in ATPS determines in which phase the reaction occurs, and this is closely related to the selection of operation units in the process design to further recover the product and recycle the phase components. Beyond the fact that enzymes were in very diluted amount in the system, they were mostly adsorbed to the bagasse after 3 h reaction in conventional hydrolysis (Figure 2). For instance, 70% of the proteins adsorbed to the bagasse in the first 3 h for the enzyme load of 20 FPU/g bagasse. Interestingly, protein desorption did not follow the glucose release profile in conventional hydrolysis at 10% WIS, regardless the enzyme load (10, 20, or 40 FPU/ bagasse) (Figure 2).

**Figure 3:**
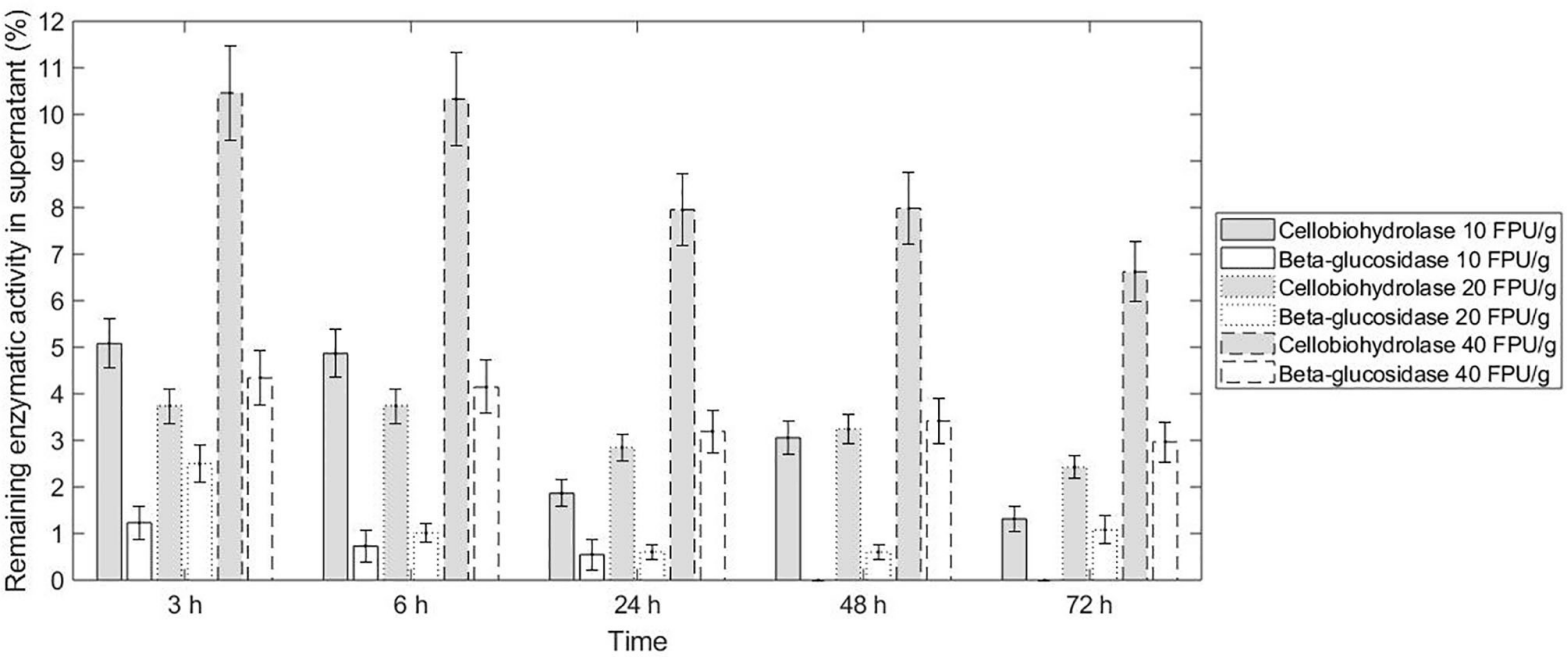
Selective adsorption of enzymes to the sugarcane bagasse during the conventional hydrolysis reaction at 10% WIS (solid load), at different enzyme loads of 10 FPU/g bagasse, 20 FPU/g bagasse, and 40 FPU/g bagasse. The remaining enzymatic activity corresponds to the percentage of activity quantified in the supernatant in relation to the theoretical activity in case of no enzymatic adsorption. The total theoretical activities of cellobiohydrolase and β-glucosidase are, respectively, 1.7 ± 0.2 and 3.9 ± 0.5 (enzyme load 10 FPU/g bagasse), 4.1 ± 0.4 and 9.2 ± 1.2 (enzyme load 20 FPU/g bagasse), and 8.3 ± 0.8 and 18.4 ± 2.4 (enzyme load 40 FPU/g bagasse).

The two specific activities tested in the commercial cocktail presented similar adsorption to sugarcane bagasse, and this non-selective adsorption did not change according to the enzyme load. Both β-glucosidase and cellobiohydrolase presented <5% of enzymatic activity in the supernatant (not adsorbed) after 3 h hydrolysis, except for cellobiohydrolase at enzyme load of 40 FPU/g bagasse, which presented ~10% of that activity in the supernatant up to 6 h hydrolysis (Figure 3). We can assume that this reduction of measured enzymatic activity is not solely a consequence of protein deactivation because the non-adsorbed protein also reduced considerably after 3 h reaction (Figure 2).

Considering the protein adsorption and conversion profile of the three enzyme loads evaluated, the one at 20 FPU/g bagasse presented similar protein adsorption (in percentage to the total protein added) in comparison with the higher enzyme load for the same solid load (10% WIS). This fact indicates that the biomass could still adsorb proteins when the enzyme load increased from 20 to 40 FPU/g. However, the balance between adsorbed (~70%) and free enzymes was achieved and maintained from 20 FPU/g bagasse enzyme load—higher enzyme loads stills presents ~70% of adsorbed enzymes. On the other hand, the percentage of free cellobiohydrolases increased with the increase of enzyme load (20–40 FPU/g bagasse), indicating that there was an excess of this enzyme class in the reaction—celobiohydrolases are processive enzymes and should be adsorbed to the fibers to provide catalytic function once they slide along the cellulose chain to the next cleavage site as the product is released (Gupta et al., 2016). Moreover, considering the increase in conversion (~12%) when comparing the enzyme load at 20–40 FPU/g and the cost of the enzymes, the gain in hydrolysis seems not defensible. Then, the enzyme load of 20 FPU/g bagasse was chosen to conduct the ATPS hydrolysis.

### Comparison Between Conventional and ATPS Hydrolysis of Lignocellulosic Material

**Figure 4:**
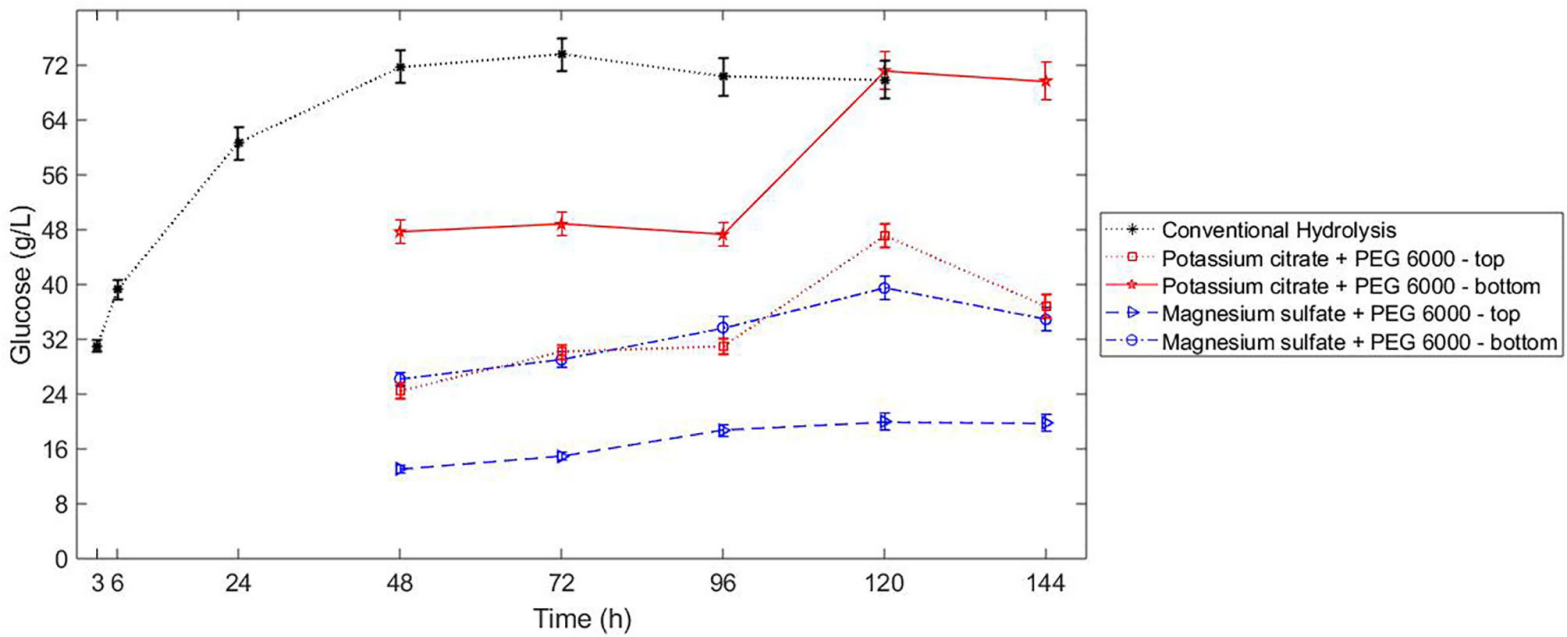
Kinetics of conventional hydrolysis of hydrothermal sugarcane (10% WIS, 20 FPU/g bagasse) bagasse vs. ATPS hydrolysis (10% WIS, 20 FPU/g bagasse), without previous liquefaction of the sugarcane bagasse.

The sugar released in bottom phase of ATPS composed by potassium citrate-PEG 6000 reached the same concentration as the conventional hydrolysis (Figure 4). Because of the uneven partition of sugars in the ATPSs composed by potassium citrate-PEG 6000 (TLL 15.3%) and magnesium sulfate-PEG 6000 (TLL 30.5%), the concentration of sugars during enzymatic hydrolysis contributed to different reaction rates in each phase of the system. For both ATPS studied, the partition coefficient of sugars was smaller than 1 (higher concentration of sugars in bottom phase). For the ATPS hydrolysis, the reaction did not discontinue after 48 h (Figure 4). In conventional hydrolysis, the sugar release ceased after 48 h reaction.

**Figure 5:**
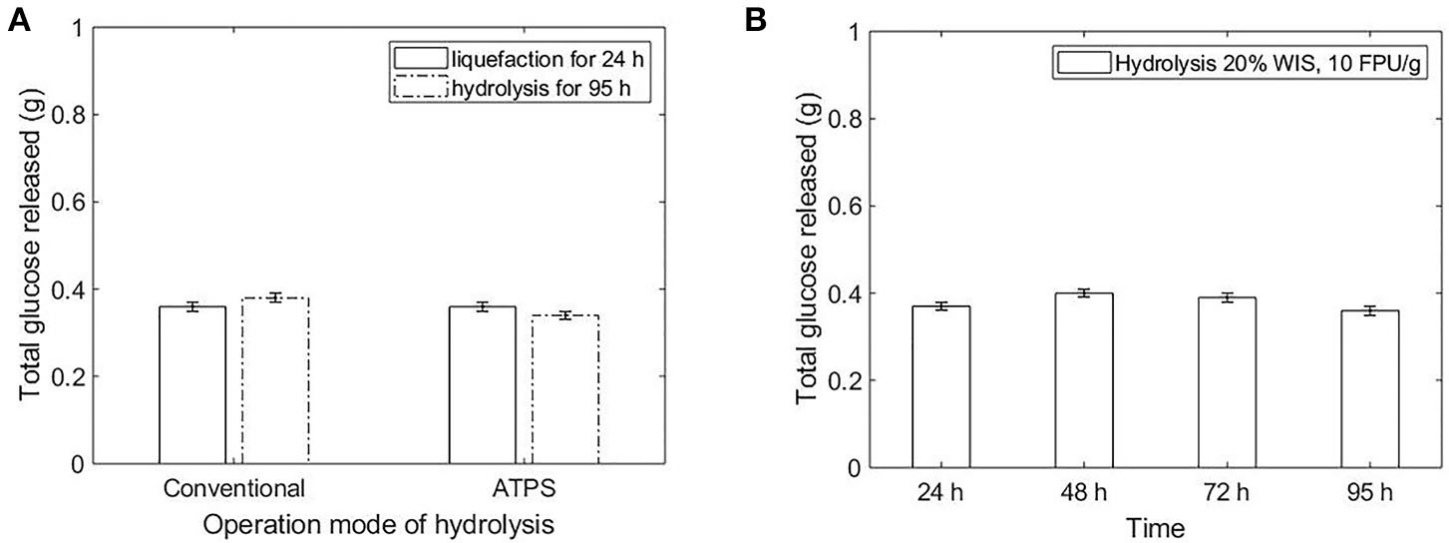
**(A)** Comparison in sugar release after liquefaction for 24 h (20% WIS solid load and 10 FPU/g bagasse enzyme load) followed by hydrolysis (total reaction of 95 h), for conventional and ATPS operation modes. **(B)** Conventional hydrolysis of sugarcane bagasse (20% WIS solid load and 10 FPU/g bagasse enzyme load)—prolongation of the liquefaction up to 95 h (conversion of 34.5% ± 1). The glucose release was presented in total grams in the reaction, in order to compare systems with different volumes.

When the system reaction was composed of liquefied substrate (sugarcane bagasse hydrolyzed for 24 h at conventional hydrolysis), no extra glucose was released for both conventional and ATPS hydrolysis (Figure 5A).

### Potential of Enzyme Recycling and Sugar Recovery in ATPS

**Figure 6:**
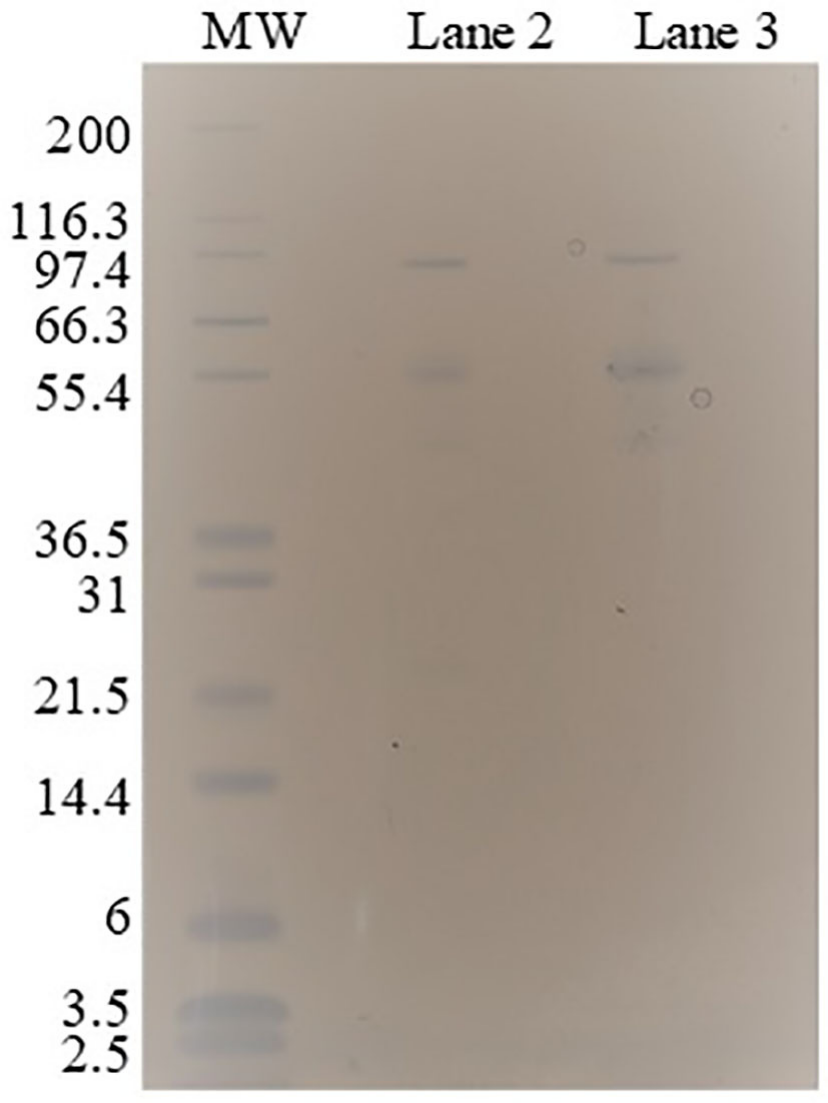
SDS–PAGE electrophoresis of enzymatic cocktail (lane 2) and bottom phase (lane 3) after partition in the ATPS 2 (potassium citrate and PEG 6000, TLL 15.3%). Molecular weights (*MW*), in kDa, in decreasing order (lane 1): 200, 116.3, 97.4, 66.3, 55.4, 36.5, 31, 21.5, 14.4, 6, 3.5, and 2.5.

Enzymes contribute in a great extent to the costs and to the conversion efficiency of a continuous process of hydrolysis (Torres, 2016). In the process configuration applied to the potassium citrate system (Figure 1B), in which top phase is the reactive phase, the free enzymes can be recovered in an ultrafiltration step. This operation unit also intends to recycle phase forming components (PEG) and recover the product (sugar). However, the partition of specific enzymes of the cocktail should be even among the phases or the entire cocktail should be one-sided partitioned to avoid the enrichment of the enzymatic cocktail with a specific activity (class of protein) in a continuous process with recycling. The ATPS 2 (potassium citrate and PEG 6000, TLL 15.3%) accomplished these requirements since all bands of proteins identified in the enzymatic cocktail (lane 2) were also present in the bottom phase (lane 3) (Figure 6).

**Figure 7:**
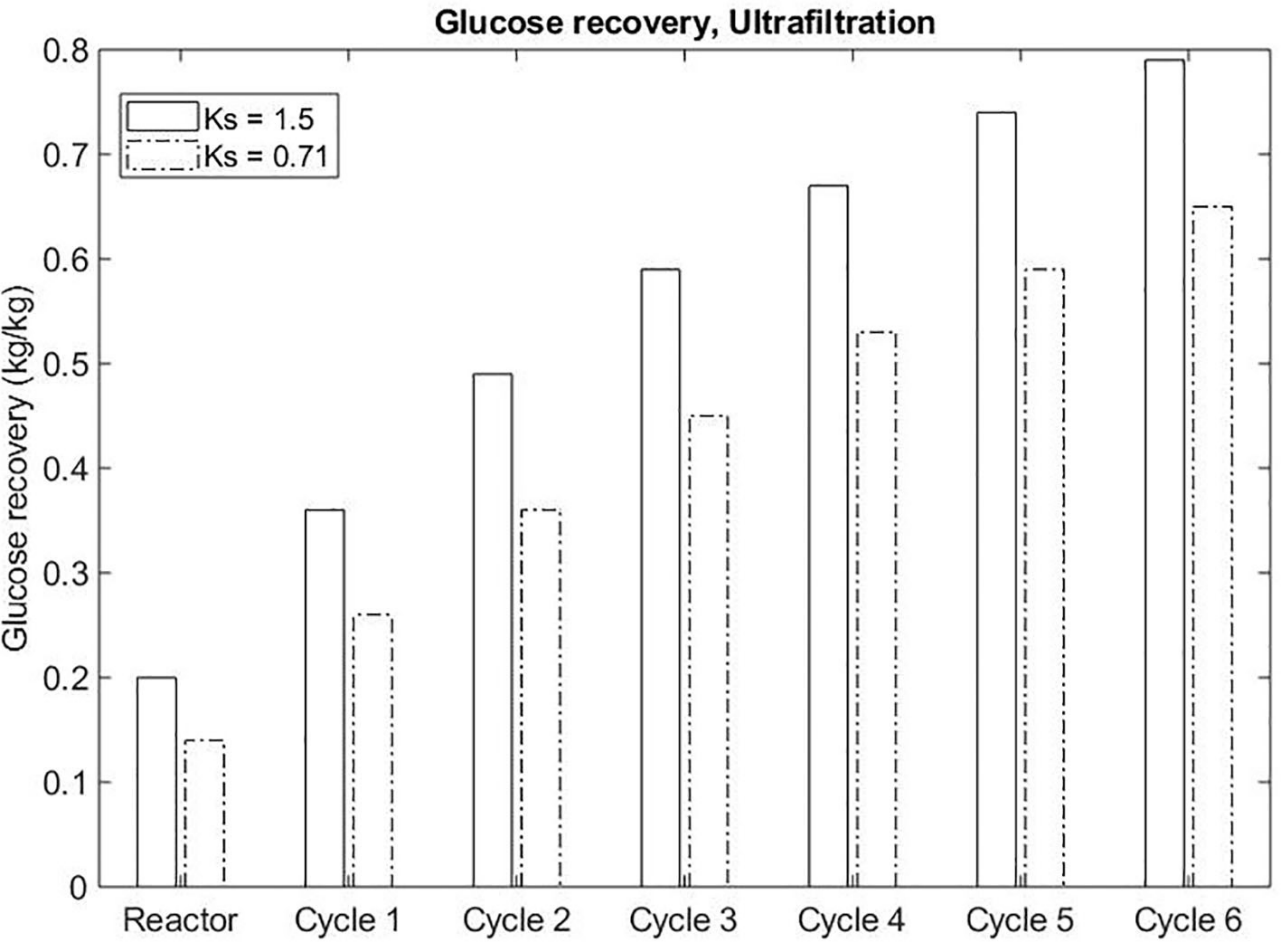
Sequential ATPS formation to recover sugar in the ultrafiltration unit of operation.

For both process alternatives, the final product (glucose) is suggested to be recovered from the polymer enriched-phase. The recovery of sugar from salt enriched-phase is rarely reported in literature. On the other hand, the separation of glucose and polymer is widely acquired through ultrafiltration. Even though some systems separate glucose preferably to the bottom phase (salt enriched-phase), the recovery from the top phase is possible through the repartitioning of the sugar-enriched bottom phase to a new sugar-depleted top phase. Evidently, the higher the partition coefficient of glucose in the system, the lower the number of cycles to achieve the desirable concentration of sugar in the end stream of the process. The efficiency of this strategy was assessed theoretically. A recovery of 0.65 kg/kg glucose was achieved through a sequential partition of glucose to the top phase for an ATPS presenting *K*_*sugar*_ of 0.71 after six cycles. If the *K*_*sugar*_ increased to 1.5, 0.67 kg/kg recovery would be achieved after four cycles (Figure 7).

## Discussion

This section discusses how the experimental results assisted the definition of parameters to a future process design. Moreover, the discussion also indicates the suitability of conducting enzymatic hydrolysis in ATPS.

### ATPS and Enzymatic Hydrolysis of Sugarcane Bagasse

In order to operate lignocellulosic hydrolysis in ATPS, the system formation and equilibrium, influence of viscosity on mixing, and analytical techniques are of paramount importance to a proper reaction conduction and quantification. Even though we showed that the bagasse has non-selective adsorption toward the phase forming components in comparison to water, we suggest the addition of bagasse to the heterogeneous solution under mixing, providing contact to all components at the same time.

During the hydrolysis, the cleavage of specific sites of the polymer chain can affect its hydrophobicity, and consequently its partition in the system. This statement can be supported by the work of Fu et al. (2019), who suggested a method to quantify hydrophobicity based on the partition of natural organic matter on ATPS composed by PEG and potassium citrate. Additionally, the density of the substrate decreases during the hydrolysis, diminishing the gravity force in favor of setting down the substrate components, which might also explain the different bagasse partitioning before and after hydrolysis.

The increase of viscosity due to high solid load can impact the mixing and consequently the efficiency of the reaction (Rosgaard et al., 2007). When operating in ATPS, the concentration of phase-forming components, specially the polymer, becomes a great contributor to increase viscosity. Then, the solid load cannot be considered as a unique factor to the mass transfer limitation (set at a maximum of 20% WIS to ATPS hydrolysis by this work). Another limitation of ATPS application relies on the analytical techniques to quantify proteins (González-González et al., 2011; Silvério et al., 2012; Glyk et al., 2015). Some strategies have already been suggested in literature using conventional methods of quantification (Bussamra et al., 2019).

Although ATPS seem to maintain the conversion rate due to the inhibitor partition in the system, the ATPS should provide a proper environment to preserve the enzymatic activity. The performance of the enzymes when incubated in the salts potassium citrate (10% w/w pH 5) and magnesium sulfate (10% w/w pH 5) was reduced ~50% in relation to the enzymatic activity in optimum conditions (conventional hydrolysis—citrate buffer 50 mM) (Bussamra et al., 2019). Although temperature can be easily controlled to operate in the optimum for the enzymes, pH is an important factor when dealing with ATPS since the different salts present different properties under pH variation, which can also impact the enzymatic activity. Therefore, an ATPS composed by harmless phase forming components to the enzymes would provide the environment to both preserve the enzymatic activity and overcome product inhibition (through sugar extraction to the opposite phase of the reaction). Alternative phase-forming components can provide optimal environment to the catalytic activity, such as ionic liquids (Ferreira et al., 2018).

### Conventional vs. ATPS Hydrolysis

Even though the product inhibition was not identified as a cause to decrease cellulose hydrolysis by Bommarius et al. (2008), other authors have refereed to product inhibition as the main factor to decrease the conversion rate at high conversions (Xiao et al., 2004; Bezerra and Dias, 2005). A previous study has identified the advantage to remove inhibitor (sugar) from the reaction in order to maintain the conversion rate (Yang et al., 2011). Here, the stagnation in sugar release after dilution of the 24 h liquefied bagasse indicates that sugar concentration is not the only factor contributing to the product inhibition in enzymatic hydrolysis (Figure 5B). Considering that 90% of the enzymes were already adsorbed to the fibers at 24 h of reaction at the enzyme load of 10 FPU/g bagasse, the reaction did not stop because of enzyme dilution and consequent inaccessibility to the fibers. Diluting the reaction system without the removal of products kept the inhibitor-to-enzyme ratio constant. Xiao et al. (2004) showed that the decrease of this ratio is crucial to overcome product inhibition. To a certain extent, the ATPS also decrease this ratio in one of the phase system, partitioning the sugar between the top and bottom. Moreover, the cellulose chain could be inaccessible after 24 h due to the increased proportion of highly-recalcitrant region of the fibers (crystalline cellulose), since amorphous regions are hydrolyzed first.

Although the mechanism of cellulase binding on lignocellulosic biomass (substrate-enzyme interaction) is not completely understood, some models can elucidate factors affecting the enzyme rates and activities (Bansal et al., 2009). Cellobiose, released during the cleavage of the bagasse chain during hydrolysis, is a non-competitive inhibitor to cellulases (Bezerra and Dias, 2005). However, some studies classify cellobiose as a secondary inhibitor once the high glucose concentration (mainly inhibitor) leads to the accumulation of cellobiose. The degree of inhibition caused by glucose is also more pronounced to cellulase mixture than to isolated β-glucosidase (Xiao et al., 2004). The reported nature and degree of glucose inhibition might be a likely cause associated with the decrease in conversion rate after 24 h conventional hydrolysis at solid load of 10% WIS and enzyme load of 10 FPU/g bagasse (Figure 2).

In the potassium citrate-PEG 6000 ATPS hydrolysis, the cellulose conversion increased in 51.5% from 96 to 120 h, whereas the conventional hydrolysis did not present any sugar release in the same period. However, similar conversion increase (54%) already happened from 6 to 24 h in conventional hydrolysis. A similar glucan conversion achieved at different times has been demonstrated when the substrate presents change in crystallinity (Gao et al., 2013). The phase forming components could influence the exposure of the amorphous part of the cellulose, as well as reducing the hydrophobicity of the cellulose surfaces, impairing the enzymes binding to the substrate. Moreover, decreasing rates of hydrolysis with high degrees of conversion have been associated to the jamming effect, which is the interference of adjacent enzymes adsorbed on cellulose surface at high protein concentration (Bommarius et al., 2008). Protein degradation could also trigger the discontinuation of the enzymatic conversion (Bansal et al., 2009).

Even though the sugar released in bottom phase during the hydrolysis conducted in ATPS composed by potassium citrate-PEG 6000 (TLL 15.3%) was similar to the sugar released in conventional hydrolysis, some peculiarities of the different approaches can benefit the implementation of the ATPS conversion. In ATPS, the inhibitor is partitioned in the system and the maintenance of the enzymatic activity after 48 h reaction for ATPS hydrolysis can be an indication that sugar in being removed from the reactive phase. However, the partitioning of sugar and consequent preservation of the reaction rate could also imply a delay in achieving the inhibitory concentration of products in the respective phase.

In both approaches (Figure 1), glucose and protein partition predominantly to the bottom phase. Even though the selectivity for sugars was higher in ATPS 5 (Table 3), the sugar release in that system was lower in comparison to ATPS 2 (Figure 4). This can be explained by the reactive phase (bottom phase, due to bagasse partition) presenting the higher concentration of inhibitor (glucose) among the phases. Moreover, ATPS 2, composed of potassium citrate, could better maintain the optimal catalytic pH than ATPS 5.

The TLL influences directly the hydrolysis performance once the high concentrated systems can decrease the enzymatic performance and limit the mass transfer of the system. On the other hand, low TLL does not present a satisfactory difference between the partition coefficients of sugar and proteins. To overcome this issue, a liquefaction of the bagasse in buffer (conventional hydrolysis) prior to ATPS formation would promote the adsorption of the enzymes to the bagasse and the conversion at high initial rates. After the ATPS formation, the sugar concentration would partition in the system and the reaction rate would be maintained until the achievement of the inhibitory concentration of products in one of the phases. The mass transfer limitation should be smaller in this mode of operation, once the substrate would be partially hydrolyzed when the ATPS are formed. Moreover, the conventional and mild environment used to liquefy the bagasse would prevent the enzymatic activity reduction due to salt contact (present in ATPS) in the beginning of the reaction. Surprisingly, the liquefaction for 24 h followed by hydrolysis did not contribute to the maintenance of the conversion rate (Figure 5). After 24 h liquefaction (conventional hydrolysis at 20% WIS solid load and 10 FPU/g bagasse enzyme load), the enzymes were already inhibited (Figure 5B). In order to take advantages of both conventional and ATPS hydrolysis, the liquefaction should provide conversion at high initial rates and the ATPS formation should enable a high total sugar release due to the partition of product to other phase. Then, a liquefaction of the bagasse for 6 h prior to hydrolysis in ATPS would provide both benefits. Moreover, a higher initial rate could be achieved by increasing the enzyme load to 20 FPU/g bagasse (Figure 2). The liquefaction before the ATPS formation also overcomes the challenge of fitting the volume of bagasse in the volume of the reactive phase, since the substrate will be partially converted and the solid load decreased when biphasic system is formed.

### Recycle and Recovery of System Components

The strategy designed to recuperate the product (sugar) in this process suggests the sequential partition of sugar to the top phase and the recovery occurring in that phase (PEG-enriched phase) after ultrafiltration. Theoretically, the permeate stream containing the product also presents soluble lignin and salt in the same concentration of the salt in top phase. The recovery of sugar could be improved by an ATPS presenting higher partition coefficient of glucose and by strategies to remove salts in accordance to the application of product stream.

In the system configuration of top phase as reactive phase, the ultrafiltration operation unit also grants the recycle of PEG and enzymes back to the process. However, enzymes do not desorb from bagasse after maximum conversion is achieved in conventional hydrolysis (Figure 2), impairing their recycle to the system reaction as free enzymes. A similar high binding capacity of around 85% of the cellulolytic enzymes has been reported by Gao et al. (2013). This irreversible cellulose binding was observed for hydrolysis involving lignocellulosic substrates, contrarily for amorphous cellulose hydrolysis, in which the substrate favors the desorption after depletion of substrate (Gao et al., 2013). However, due to the extensive solubilization of cellulose and hemicellulose, the irreversible binding of cellulose is mostly related to the presence of lignin. Moreover, the binding capacity of the enzymes does not indicate an improved cellulose conversion since the enzymes can be non-productively bound to the substrate (Gao et al., 2013). The recycle of enzymes from the supernatant has already been demonstrated to succeed at industrial conditions (high solid load and low enzyme dosage) for wheat straw substrate hydrolysis (Haven et al., 2015). This recycling strategy is restricted to the enzymatic stability of the recovered enzymes. Improved conditions for enzyme stability can enhance the potential of recycling free/desorbed enzymes (Haven et al., 2015). In a continuous system, the free enzymes are constantly removed from the reaction. The removal of desorbed/free enzymes does not impact significantly the rate and extent of the hydrolysis (Hu et al., 2018). However, it is important that the recycle occurs after the equilibrium in enzymatic adsorption is achieved.

Considering the reactive phase as the bottom phase, the enzymes are recycled back to the system adsorbed to the unconverted biomass. The recycle of enzymes associated with insoluble solids has already been proven as an effective method to decrease enzyme usage when operating hydrolysis in several rounds (Weiss et al., 2013). The bound enzymes are capable of hydrolyzing cellulose from fresh substrate. Moreover, the solid recycle method increases the contact time between catalyst and substrate, and enables unreacted substrate to return to the reaction. Increasing the amount of solid fraction recycled also increases the glucose release. However, the recycle of solids leads to increased lignin in the solid composition and increased total solid concentration, which requires higher tank size and a process able to deal with higher solids concentration and volumes. Thus, the process should be balanced between recycling enzyme activity and increased operating solids concentration. This method of recycling enzymes requires complementation of fresh enzymes at each recycle round once there are losses of enzymes in the liquid fraction and non-productive binding to lignin (Weiss et al., 2013).

### Parameter Definition to a Process Design and Technical Challenges

Here, we bring an overview of parameter definition when designing the extractive process to relieve product inhibition based on ATPS. In addition to the process definition provided by the experimental data, we indicate other aspects of the process design that could be benefited by additional experiments (summarized in Table 4).

**Table 4:** Information to be retrieved from experiments to provide empirical basis to a reliable definition of parameters to the process design.

**Information substantiated by experiments**	**Type of parameters to be defined in the process design**
Partition of specific enzymatic classes after ultrafiltration (retentate and permeate)	Feasibility to recycle enzymes in terms of enrichment of one specific enzymatic class in continuous operation
Enzymatic activity in the ultrafiltration unit (feed and retentate). Model: potassium citrate-based ATPS	Feasibility to recycle enzymes in terms of recovery efficiency (retention coefficient for the process design)
PEG concentration in the ultrafiltration unit (feed and retentate). Model: potassium citrate-based ATPS	Feasibility to recycle PEG (concentration factor for the process design)
Partition of glucose in ATPS (top and bottom) after ultrafiltration, in which retentate composes the next top phase for the repartition of glucose in ATPS	Number of ATPS cycles to repartition glucose and achieve the desired sugar recovery
Thermodynamic model to predict phase separation and partition of solutes in ATPS	Contribution to a reliable calculation of phase composition and partition coefficient of solutes at different TLL (after recycle of PFCs)

Because the phase-forming components can influence the partition of the bagasse in the system, polymer and salts can be chosen in order to promote the reactive phase (enriched in substrate and adsorbed enzymes) apart from the glucose-enriched phase. Moreover, the selection of phase forming components and concentration (TLL) should also be based on the capacity of maintaining the enzymatic activity of cellulases. The appropriate choice of components determines the efficiency of the ATPS-based hydrolysis. As presented in Figure 1, the ATPS reactor has a separate vessel for phase separation. At this stage, the bagasse is partially hydrolyzed and the process design is not affected by the different partitioning of bagasse observed before and after hydrolysis.

In a continuous process involving ATPS, the recycling of components requires more attention than monophasic systems (conventional hydrolysis) since the recycled stream might not contain the same composition of phase forming components as the initial one (the TLL can alter at each cycle) depending on the recycled, purged, and fresh streams of system components. In order to estimate the outlet concentrations of top and bottom phases, given the new inlet composition of the ATPS, phase diagrams previously reported by Bussamra et al. (2019) were used. To automatically predict the new phase compositions partition coefficients, a thermodynamic model should be developed and implemented on the design calculation. Different compositions of top phase can affect the repartitioning of sugar. Giving the experimentally determined partition coefficient of this solute in the corresponding system composition, the authentic number of cycles to repartition glucose and achieve the desired sugar recovery can be empirically determined in order to model a reliable recovery operation.

Although the conventional hydrolysis does not indicate a desorption of enzymes after the reaction, PEG has been demonstrated to favor enzyme adsorption on the surface of the biomass, preventing the binding to the lignin (Malmsten and Van Alstine, 1996; Haven and Jørgensen, 2013). This feature of the ATPS could promote the recycle of enzymes and validate the hydrolysis model proposed by Stickel et al. (2018), in which the concentration of free enzymes increases with the decrease of substrate (glucan). Moreover, the non-selective partition and adsorption of the different enzymatic activities tested suggest a constant recycled cocktail composition. In the dextran-PEG 20000 system reported by Tjerneld et al. (1985), all the cellulolytic enzymes partitioned to the bottom phase, not affecting the continuous process of cellulose conversion in ATPS (Tjerneld et al., 1991). The paradigm that β-glucosidase (from Cellic CTec 2) would not adsorb to the substrate or only adsorb to a min or extent had already been broken by Haven and Jørgensen (2013). Some preferential adsorption of enzymatic classes has been observed for the cocktail Cellic CTec 3, in which cellobiohydrolase presented a higher binding affinity toward the substrate than endoglucanase (Hu et al., 2018). The preferential adsorption can be connected to the required enzymatic classes to hydrolyze a specific substrate composition. However, the recycled stream of the enzymes is an outcome not only from the ATPS separation reactor, but also from the ultrafiltration operation unit. Based on that, the evaluation of the partition of specific enzymatic classes on retentate and permeate from ultrafiltration should also be considered. Consequently, the recovery efficiency should be assessed in terms of maintenance of enzymatic activity after ultrafiltration (retentate stream), and considering the number of ultrafiltration cycles needed to repartition glucose and obtain the desirable recovery of this product. When assessing the enzymatic activity of the retentate to be recycled to the system, it is also important to regard the theoretical loss of enzymes in the permeate and purge streams (Stickel et al., 2018).

Combining the empirical information reported in this work (Table 1) with literature evidence, a qualitative process was designed to conduct enzymatic hydrolysis in ATPS. Moreover, the kinetic data on conventional and ATPS enzymatic hydrolysis generated here can improve the fundamental understanding of variables regarding the enzymes and substrate. A more detailed and phenomenological understanding of the hydrolysis process can give insights on kinetic models and/or validate existent ones (Bansal et al., 2009). In conclusion, the set of information acquired so far indicates that the ATPS hydrolysis could be reasonable modeled under such considerations.

## Conclusion

This study demonstrated the suitability of conducting the enzymatic hydrolysis of lignocellulosic materials in aqueous two-phase systems (ATPS). This extractive technique, when applied to enzymatic conversions in the presence of solid substrate, presents peculiarities regarding the enzymatic activity performance and the partition of the substrate in dependence on the system composition. In the ATPS enzymatic hydrolysis, the reactive phase is determined by the substrate enriched-phase since the majority of the enzymes adsorbs to the fibers. The process design for such application involves a liquefaction of the bagasse prior to the ATPS hydrolysis. Because the proteins do not desorb from the fibers along the hydrolysis, the approach considering the recycle of enzymes when adsorbed to the substrate is more appropriate. Experimental studies on the ultrafiltration unit operation would substantiate the assumptions regarding the feasibility to recycle not adsorbed enzymes and phase forming components, and to recover the product. Based on the data acquired and literature evidence, this work provides valuable information (technical conditions) to a future quantitative evaluation of the processes.

## Data Availability Statement

All datasets generated for this study are included in the article.

## Author Contributions

BB wrote the manuscript, participated in the design of the experiments, experiments performance, data analysis, and data interpretation. PM performed some of the experiments reported at this work, participated on the design of the experiments and discussion about the experimental results. VV contributed to the conceptual process design, defining the theoretical bases for the process design, producing the modeling results, and writing of Topic 11 in Methods. SM supervised the work, provided close revision of the results along the data generation, and assisted on data discussion. AC and LW supervised the work, contributed to the scope of the work and to the revision of the manuscript. MO supervised the work, contributed to the scope of the work, to the close discussion on each experimental data obtained, and to the revision of the manuscript. All authors contributed to the article and approved the submitted version.

## Conflict of Interest

The authors declare that the research was conducted in the absence of any commercial or financial relationships that could be construed as a potential conflict of interest.
